# Supplementary material for: Persisting effects of jaw clenching on dynamic steady-state balance
Source: PLoS One. 2024 Feb 22;19(2):e0299050. doi: 10.1371/journal.pone.0299050 (PMC10883567; doi:10.1371/journal.pone.0299050)
Supplement: S1 Table — JBT = jaw clenching and balance training, OBT = only balance training and CON = no-training control group. The results are represented as mean ± standard deviation. (PDF) [file pone.0299050.s001.pdf]

		T1		T2	
		Jaw clenching	Non-clenching	Jaw clenching	Non-clenching
TAE in s	JBT	15.8 ± 6.1	14.0 ± 5.8	21.7 ± 4.2	20.8 ± 3.6
	OBT	16.0 ± 6.0	14.9 ± 5.2	21.9 ± 3.9	21.5 ± 4.0
	CON	15.8 ± 5.9	14.3 ± 8.1	19.0 ± 6.8	18.5 ± 7.1
iEMG of TA in %	JBT	10.8 ± 5.9	11.2 ± 7.9	5.2 ± 4.0	3.9 ± 3.3
	OBT	10.6 ± 8.5	9.3 ± 7.9	4.0 ± 4.4	3.2 ± 3.1
	CON	12.0 ± 15.9	10.4 ± 11.3	6.2 ± 5.6	6.9 ± 5.5
iEMG of BF in %	JBT	3.7 ± 1.9	3.6 ± 2.3	2.1 ± 2.1	2.1 ± 2.3
	OBT	4.7 ± 3.8	4.0 ± 3.2	2.4 ± 2.3	2.4 ± 2.1
	CON	3.6 ± 2.0	4.4 ± 3.2	2.5 ± 1.6	2.7 ± 1.3
iEMG of RF in %	JBT	7.4 ± 6.4	6.9 ± 5.5	4.0 ± 3.0	4.2 ± 3.3
	OBT	7.1 ± 6.6	6.6 ± 6.7	3.1 ± 3.0	2.8 ± 3.0
	CON	6.6 ± 5.4	5.3 ± 3.5	4.1 ± 3.1	4.3 ± 3.5
iEMG of GM in %	JBT	6.7 ± 6.5	5.8 ± 6.1	4.2 ± 4.7	4.2 ± 5.5
	OBT	7.9 ± 8.7	7.3 ± 6.8	2.9 ± 2.8	3.2 ± 2.6
	CON	8.6 ± 6.4	8.3 ± 6.7	4.7 ± 3.3	5.5 ± 4.6
